# Global impact on metabolic capacity of yeast cell factories by optogenetic control of the cAMP–PKA axis

**DOI:** 10.1128/aem.02498-25

**Published:** 2026-05-18

**Authors:** Mohamed Watad, Jonathan Trauth, Filipp Bezold, Ammar Baker, Bastian Pook, Johannes Scheffer, Hagen Nusshär, Sophia Hasenjäger, Nicole Paczia, Lars-Oliver Essen, Christof Taxis

**Affiliations:** 1 Unit for Structural Biochemistry, Department of Chemistry, Philipps-University Marburg 54308 , Marburg, Germany; 2 Department of Biology/Genetics, Philipps-University Marburg 9377 , Marburg, Germany; 3 Max Planck Institute for Terrestrial Microbiology 28310, Marburg, Germany; 4 Department of Medicine, Health and Medical University Erfurt, Erfurt, Germany; Chalmers tekniska hogskola AB, Gothenburg, Sweden

**Keywords:** adenylate cyclase, optogenetics, Ras/cAMP/PKA pathway, proteomics, energy metabolism

## Abstract

Dynamic metabolic engineering enables temporal redirection of microbial metabolism from biomass production to product synthesis. Here, we show that optogenetic control of protein kinase A (PKA) activity via light-regulated modulation of intracellular cyclic AMP (cAMP) levels can enhance heterologous production of β-carotene and cordycepin in *Saccharomyces cerevisiae*. To enable exclusive, glucose-independent control of cAMP synthesis, the photoactivatable adenylyl cyclase bPAC from *Beggiatoa* sp. was introduced into cells lacking the endogenous adenylyl cyclase Cyr1 or with lowered Cyr1 levels using an optogenetically controlled degron. Despite being growth-competent under illumination, the bPAC-containing yeast strain showed alterations in energy metabolism under all conditions. Quantitative proteome analysis using timsTOF mass spectrometry revealed profound changes in central carbon metabolism, sulfur homeostasis, energy charge, and ribosome biogenesis upon uncoupling cAMP from nutrient-dependent regulation, particularly under sustained light activation. These results highlight the critical role of dynamic Cyr1-dependent regulation for central metabolism, and underscore the biotechnological promise of refined PKA-targeted strategies for eukaryotic cell factories.

**IMPORTANCE:**

Carbon-footprint-minimized production of fine chemicals, pharmaceuticals, and biofuels requires optimized microbial cell factories with tailored metabolic performance. We employed optogenetic dynamic metabolic engineering in baker’s yeast by uncoupling nutrient sensing from cAMP signaling using a light-controlled adenylate cyclase. Precise light regulation of intracellular cAMP levels and PKA activity enabled acute control of the metabolism, redirecting resources toward product synthesis, and boosting the production of valuable compounds such as β-carotene and cordycepin. Quantitative proteomics revealed that uncoupling of the cAMP–PKA axis from glucose sensing profoundly reprograms the central carbon metabolism and other key cellular processes. This approach provides a blueprint for refined, light-tunable strategies targeting the cAMP–PKA axis directly with light, e.g., for enhanced bioethanol production. Moreover, our data provide evidence for the profound influence of the cAMP–PKA axis on metabolism and balanced energy production that are fundamental for efficient production in microbial cell factories.

## INTRODUCTION

Sustainable production of fine chemicals, pharmaceuticals, and biofuels requires optimized microbial cell factories with tailored metabolic performance and minimal environmental impact (1, 2). Key challenges involve maximizing yield and minimizing costs while maintaining economic viability. Traditional strain engineering methods, e.g., synthetic biology, adaptive evolution, and static metabolic engineering, are laborious, requiring iterative cycles of design, assembly, testing, and optimization to turn common strains into robust cell factories for specific metabolites (3–8).

Dynamic metabolic engineering was introduced to uncouple cell growth from the production phase, either for producing toxic compounds or diverting resources to production (9). This approach necessitates an efficient, low-cost inducer suitable for large-scale fermentation (10, 11). Light is an ideal inducer: it is cheap, non-toxic, non-degradable, highly tunable, and metabolically inert in non-photosynthetic microbes (12–14). Diverse set-ups have been engineered to ensure high illumination strengths of microbial cell factories during lab-scale fermentations (15, 16).

Many optogenetic tools have emerged recently for dynamic metabolic engineering, enabling precise regulation of cellular processes, such as transcription, translation, protein stability, localization, and activity (12, 17–22). *Saccharomyces cerevisiae* is an attractive host for optogenetic dynamic metabolic engineering approaches due to its general acceptance as a safe organism, simple morphology allowing large-scale fermentations, wealth of available tools, and a deep understanding of its metabolism. These advantages are reflected in its widespread use to showcase the potential of optogenetics in metabolic engineering (23–26). Dynamic metabolic engineering aims to change substrate flux from pathways necessary for growth toward a competing pathway that generates a desired product or uses an inducer to activate the enzymes necessary for product formation; both approaches have been realized by optogenetics (9, 23, 26).

The photo-activatable adenylyl cyclase from *Beggiatoa* sp. (bPAC) is commonly used to manipulate cyclic-AMP (cAMP) levels in diverse organisms, offering tight temporal and spatial control (27, 28). In *S. cerevisiae*, bPAC has been used to investigate PKA dynamics through real-time regulation of cAMP levels (29).

The second messenger cAMP is crucial for control of protein kinase A (PKA) activity, thereby influencing gene expression, metabolic decisions, cell proliferation, and development (30–34). In budding yeast, cAMP modulates PKA activity, a master regulator of growth as well as metabolic and developmental decisions (35). Endogenous regulation of cAMP relies on the enzymatic activity of the adenylyl cyclase Cyr1 in response to extracellular glucose availability and intracellular glycolytic flux. Two molecules of cAMP bind the regulatory PKA subunit Bcy1, which releases the active catalytic kinase (isozymes Tpk1, Tpk2, and Tpk3) (36). Several transcription factors respond to PKA activity changes, thereby altering the transcriptome (37). As a result, the cAMP–PKA axis accounts for >90% of glucose-induced transcriptional responses (38). The phosphodiesterases Pde1 and Pde2 act as negative regulators of cAMP levels by hydrolyzing cAMP to AMP (39).

We investigated bPAC-induced cAMP changes and subsequent PKA modulation of energy metabolism and the proteome to increase heterologous production in budding yeast. The bPAC-dependent strain uncouples adenylyl cyclase activity and cAMP production from natural glucose sensing and glycolytic flux, making cell proliferation entirely reliant on bPAC-generated cAMP. Accordingly, we quantified cAMP levels, growth dynamics, energy metabolites, and proteome changes. We found increased production of the heterologous compounds β-carotene and cordycepin under conditions of low adenylyl cyclase activity. This shows the potential of an optogenetic metabolic engineering approach targeting the cAMP–PKA axis for biotechnology applications.

## RESULTS

**Fig 1:**
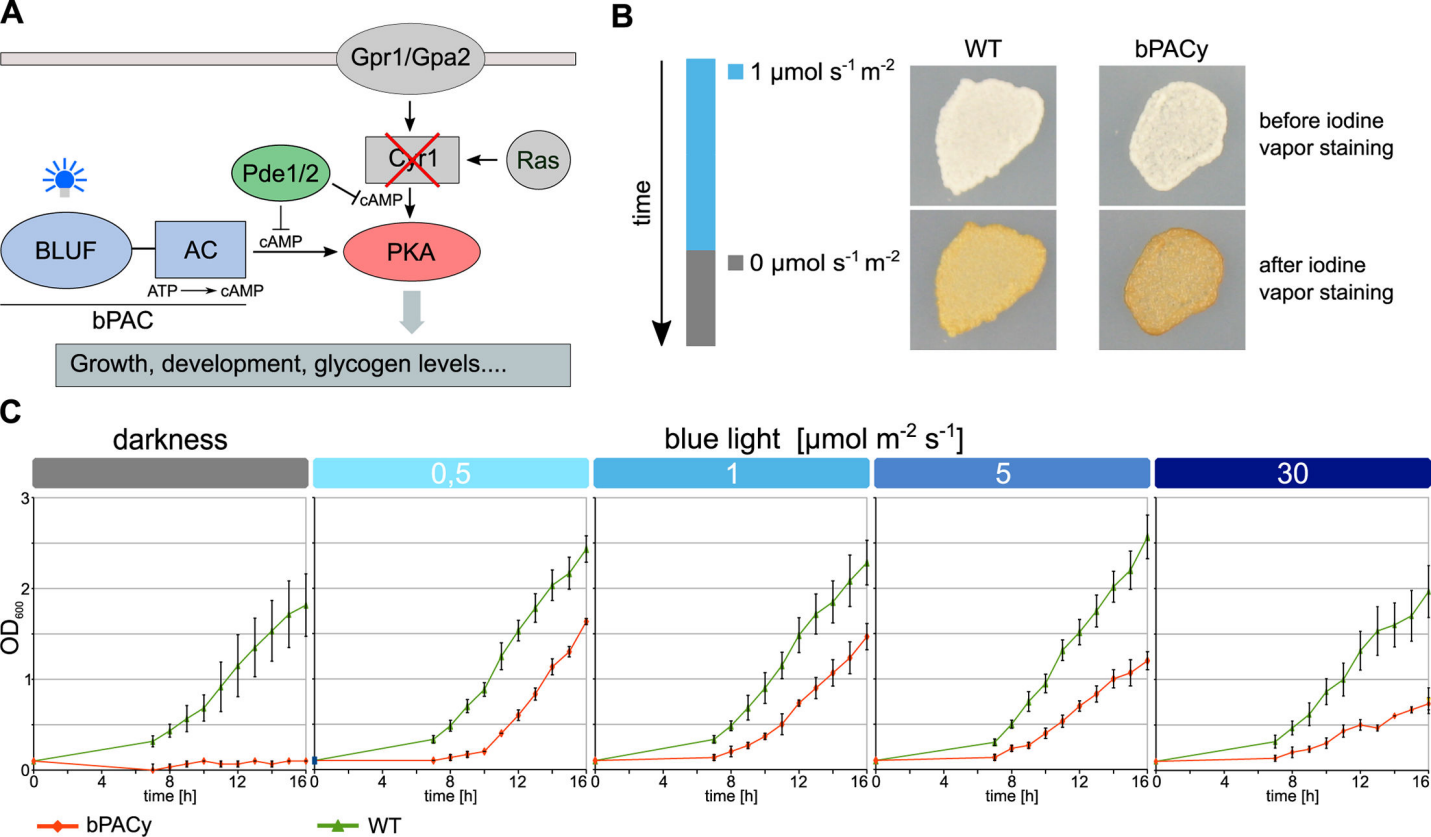
Fully light-controlled cAMP production in *S. cerevisiae*. (**A**) Scheme illustrating the isolation of PKA from nutritional signals and implementation of light control. In bPACy cells, the activity of the adenylyl-cyclase (AC) domain is regulated by a blue light using FAD (BLUF) domain, while the endogenous gene encoding the adenylyl-cyclase Cyr1 has been removed. This isolates cAMP production and PKA activity from endogenous regulatory inputs such as the Gpr1/Gpa3 module and the Ras proteins that regulate Cyr1 activity. Remaining is the influence of phosphodiesterases Pde1 and 2, which hydrolyze cAMP to AMP. (**B**) Iodine vapor staining of WT (YCR75) and bPACy cells (YSEB38). A darker color indicates lower PKA activity, resulting in higher storage carbohydrate levels. Strains were grown in patches on the same YPD-plate for 48 h with blue light illumination (1 µmol m^−2^ s^−1^) and were kept for an additional 24 h in darkness before iodine vapor staining. Pictures of the patches were taken before and after the staining procedure. (**C**) Growth curves for WT (YCR75) and bPACy cells (YSEB38) exposed to different blue light intensities as indicated. OD_600_ measurements were performed to obtain the curves (*n* = 3; error bars standard error of the mean). Cells were diluted to an OD_600_ of 0.1 into fresh LFM medium at the beginning of the experiment.

Cyclic AMP control of PKA activity affects numerous gene products and their activity, either directly through phosphorylation or indirectly and more slowly by changing the expression levels of their encoding genes. To investigate whether cAMP modulation of PKA activity by light can be used to steer the metabolism of yeast, we used a strain in which cAMP–PKA signaling is placed under exclusive optogenetic control. This strain harbors the heterologous photo-activated adenylyl cyclase (PAC) from *Beggiatoa* sp. (bPAC), while the essential gene encoding the endogenous adenylyl cyclase of yeast, *CYR1*, is deleted (Fig. 1A). Consequently, the resulting bPAC yeast strain (bPACy) should proliferate well only under blue light illumination, as cell growth strictly depends on light-triggered cAMP synthesis and concomitant PKA activation (22). For phenotypic validation of PKA activity control, we visualized glycogen by iodine vapor staining, as glycogen accumulation is negatively regulated by PKA (40). Accordingly, when kept in darkness after an initial growth period under weak blue light illumination, darker stained patches indicate higher accumulation of glycogen in bPACy cells compared to wild-type (WT) cells due to repressed PKA activity (Fig. 1B).

Quantification of WT and bPACy cell growth in liquid cultures under different light conditions revealed a complex growth phenotype of bPACy cells. The bPACy strain exhibited no observable growth in darkness, whereas WT cultures proliferated robustly irrespective of illumination conditions. Optimal growth of the bPACy strain was achieved at low fluxes of 0.5 and 1 µmol m^−2^ s^−1^, with a modest reduction at 5 µmol m^−2^ s^−1^ and severely impaired proliferation at high flux, i.e., 30 µmol m^−2^ s^−1^ blue light photons (Fig. 1C). Overall, the absence of illumination did not sustain growth of bPACy, arguing for insufficient cAMP production, whereas excessive, non-physiological levels of cAMP, as generated by high light flux, apparently impair growth.

**Fig 2:**
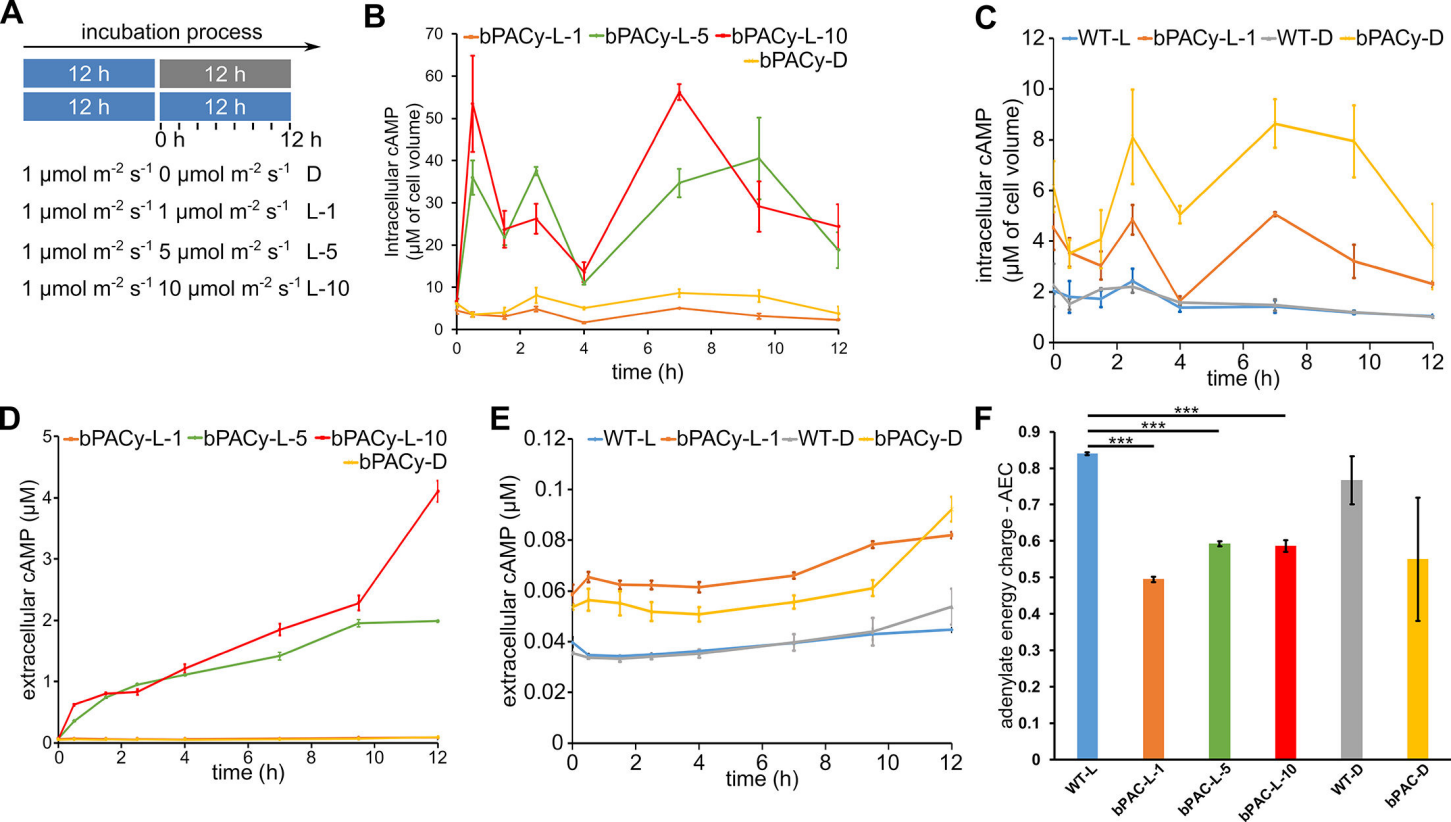
(**A**) Scheme illustrating the illumination regimen to measure intra- and extracellular cAMP levels. Cells were kept in the same medium during the 24 h of incubation. (**B**) Intracellular cAMP concentrations in bPACy (YSEB38) cells incubated in darkness and at light fluxes of 1, 5, and 10 µmol m^−2^ s^−1^ blue light (465 nm; bPACy-D; bPACy-L-1; bPACy-L-5; bPACy-L-10). (**C**) Intracellular cAMP concentrations in WT (YCR75) and bPACy (YSEB38) cells incubated in darkness (WT-D; bPACy-D) or at 1 µmol m^−2^ s^−1^ blue light (465 nm; WT-L-1; bPACy-L-1). (**D**) Extracellular cAMP concentrations in the medium of the strains used in panel B. (**E**) Extracellular cAMP concentrations in the medium of the strains used in panel C. (**F**) The adenylate energy charge (AEC) was calculated to assess the cellular energetic state. The formula (ATP + 0.5⋅ADP)/(ATP + ADP + AMP) was used to calculate the AEC. (**B–F**) Mean values of three independent biological replicates are shown; error bars indicate standard deviation.

The bPACy strain enabled us to examine how variations of blue light intensity translate into variations of intracellular cAMP levels. For that, we designed a two-phase cultivation protocol: an initial 12-hour growth-promoting period under low-intensity blue light (465 nm, 1 µmol m^−2^ s^−1^) was followed by 12 h of either darkness or continued incubation at varying light fluxes, with regular sampling of cells and culture supernatant (Fig. 2A). This cultivation protocol was necessary to obtain enough cells for metabolomic and proteomic analyses. Intracellular cAMP levels in the bPACy strain responded dynamically to illumination over time, achieving up to 55 µM at high fluxes of 10 µmol m^−2^ s^−1^, whereas cAMP in WT cells remained mostly constant around an average concentration of 2 µM, regardless of incubation in darkness or under 1 µmol m^−2^ s^−1^ of blue light photons (Fig. 2B). During the lag phase, i.e., within the first 4 h of growth after illumination change, cAMP levels in bPACy cells underwent some fluctuations that were more pronounced than in the WT strain. Unexpectedly, intracellular cAMP levels of bPACy cells exposed to a low flux of 1 µmol m^−2^ s^−1^ photons, resulting in 2–5 µM cAMP, were consistently lower by roughly 50% than in bPACy cells kept in darkness (4–8 µM, Fig. 2C). To account for this counterintuitive discrepancy, we also assessed extracellular cAMP concentrations in the medium, as a pool of extracellular cAMP may affect intracellular levels by a transport process into the cell. Indeed, we found high cAMP levels of up to 4 µM at a photon flux of 10 µmol m^−2^ s^−1^ in the medium of bPACy cultures. These cAMP levels in the medium of bPACy cells exposed to higher light fluxes were steadily rising over time (Fig. 2D). At low flux (1 µmol m^−2^ s^−1^ photons), cAMP amounts in bPACy medium were already twice as high as in WT medium (Fig. 2E) and elevated by ~20% compared to bPACy cells in darkness. At 12 h of incubation, the intracellular cAMP level of bPACy cells in darkness dropped by a factor of two (Fig. 2C), with a concomitant rise in the extracellular cAMP level (Fig. 2E), hinting at a loss of intracellular cAMP to the extracellular pool. Summing both pools resulted in mostly stable total cAMP levels over time in WT cells and slightly fluctuating, as well as slightly increasing, cAMP levels in the bPACy cells (Fig. S1A). In accordance with the intracellular and extracellular cAMP levels, we observed growth of WT and bPACy cells under all conditions during the experiment, even in the bPACy strain shifted to darkness, although at a much lower rate than in the WT strain (Fig. S1B). A viability staining after the 12-hour time point did not reveal pronounced cell death in the bPACy strain exposed to darkness (Fig. S1C). The difference in viability between the WT and bPACy strains was 10% and was not found to be statistically significant.

Given the uncoupling of nutrient response from cAMP due to the Δ*CYR1* background, we also quantified intracellular and extracellular concentrations of ATP, ADP, and AMP. After the lag phase, intracellular ATP, ADP, and AMP levels changed differently between the strains (Fig. S1D through F). In WT, ATP was rising to ~1.5 mM after 12 h with concomitant drops of ADP and AMP to 500–700 µM and 100–300 µM, respectively. In the bPACy strain, ATP concentrations were consistently lower than in WT (roughly 50%), but roughly two-fold higher for AMP after 12 h.

The adenylate energy charge (AEC), defined as ([ATP] + 0.5[ADP]) / ([ATP] + [ADP] + [AMP]) (41), indicates the cellular energy status, with values of 0.8–0.9 typical of growing yeast (42). Within the first hours of condition change, we found a strong fluctuation of the AEC in the bPACy and WT strains, with AECs getting as low as 0.2–0.3, which recovered after 12 h to values <0.6 for bPACy, while that of the WT strain was raising to ~0.8, consistent with normal growth behavior (Fig. 2F; Fig. S1G). AEC values of <0.6 are known to indicate yeast cells starved of their primary carbon source, for example, directly after a diauxic shift, while values of <0.3 have been predicted to cause metabolic instability (43) and are otherwise inducible in yeast only by transient starvation or PKA suppression. In bPACy cells at all investigated illumination conditions (darkness, 1, 5, 10 µmol m^−2^ s^−1^ blue light), the lowered AEC, ATP/AMP ratios of <1, and an absolute ATP concentration of <1 mM imply metabolic stress due to cAMP/glucose uncoupling by exchanging Cyr1 for bPAC. The lowered AEC values for bPACy explain the growth impairment of this strain compared to the WT, even under optimal illumination conditions (Fig. 1C).

In contrast, extracellular ATP and ADP levels (~40 nM, <10 nM) were indistinguishable between WT and bPACy cells when exposed to low blue light fluxes (1 µmol m^−2^ s^−1^, Fig. S1E and F). Notably, after 12 h, the dark-arrested bPACy strain showed enhanced leakage for all adenylyl species, as noted before for cAMP (Fig. S1D through F), while extracellular AMP levels increased only slightly in the medium of WT cells under both conditions and in the medium of bPACy cells exposed to low photon flux (Fig. S1F). In bPACy cells exposed to higher light fluxes (5 and 10 µM m^−2^ s^−1^), intracellular ATP and ADP levels were somewhat fluctuating, whereas extracellular levels decreased and then increased again (Fig. S2A and B). Intracellular AMP levels decreased over time, whereas extracellular AMP levels increased over time (Fig. S2C).

Overall, the bPACy strain allows robust growth under low to medium light flux conditions, despite showing an energy-starved metabolic state indicated by a lowered AEC during the observation period. Apparently, the ability of *S. cerevisiae* to respond dynamically to nutrient changes, e.g., during a diauxic shift from glucose to ethanol, allows this organism to tolerate a wider range of AEC for growth.

**Fig 3:**
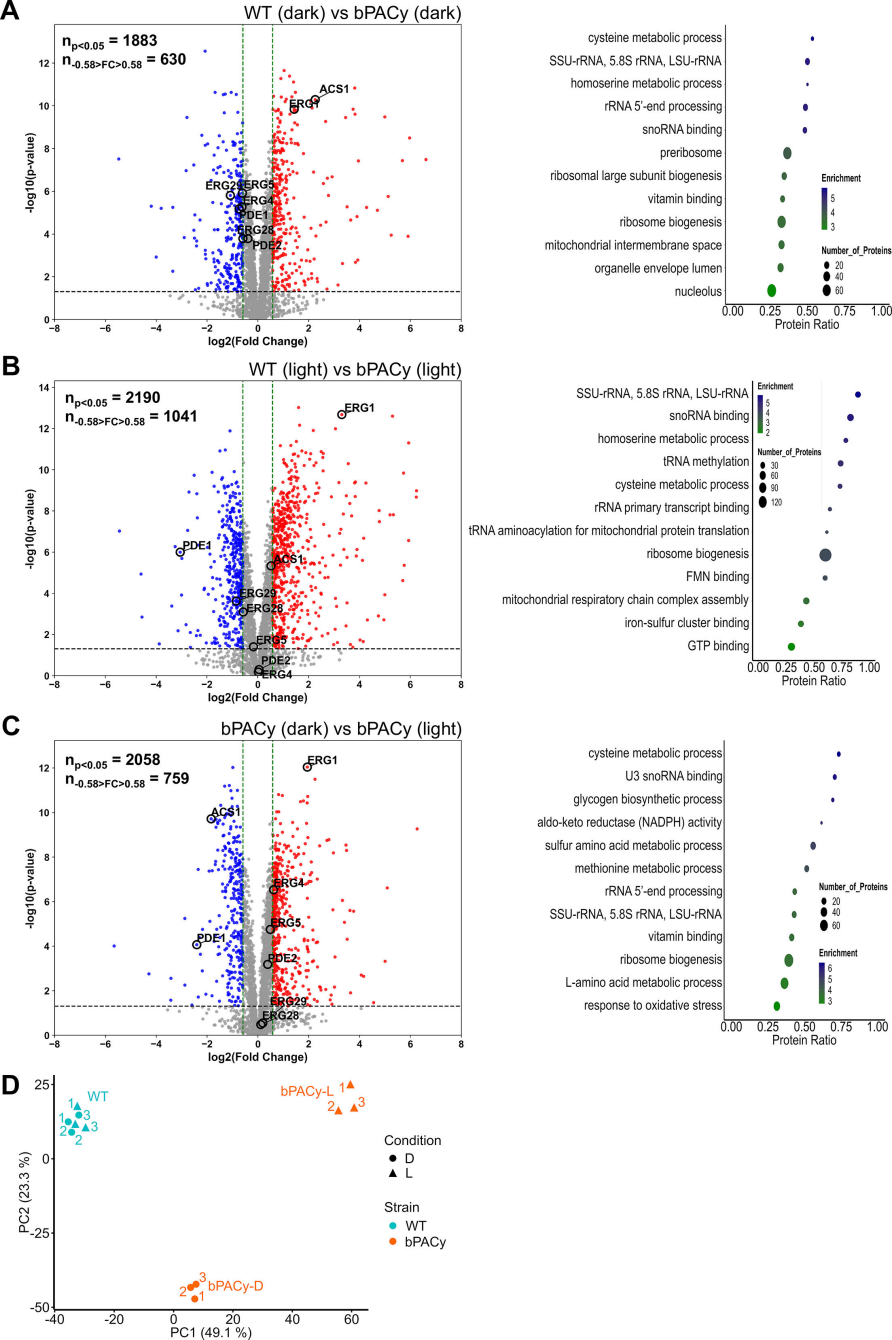
Proteomic profiling of WT and bPAC-expressing S. cerevisiae under light and dark conditions. Bottom-up proteomics experiments were performed using timsTOF Pro on protein extracts from WT and bPACy cells grown in LFM medium at 30°C and 80 rpm. Cultures were pre-grown for 12 h under blue light (465 nm) at a light flux of 1 μmol m⁻² s⁻¹ and maintained under continuous illumination for an additional 12 h (WT light, bPACy light) or shifted to darkness for 12 h (WT dark, bPACy dark), in both cases resulting in a total cultivation time of 24 h. Differentially expressed proteins were identified by volcano plot analysis (cutoffs: −0.58 > log₂FC > 0.58, *P* < 0.05). For each comparison, the volcano plots (left) display the distribution of protein fold changes, with significantly upregulated proteins highlighted in red and downregulated proteins in blue, while the corresponding gene ontology (GO) enrichment analyses (right) summarize the functional categories most affected. GO analysis was conducted using GOATOOLS in Python (criteria: −0.58 > log₂FC > 0.58, *P* < 0.05, p_fdr_bh < 0.01) and visualized in R. (**A**) Proteome changes between WT (dark) and bPACy (dark). (**B**) Same as panel A for WT (light) and bPACy (light). (**C**) Same as panel A for bPACy (dark) and bPACy (light). (**D**) Principal component analysis (PCA) of proteomic profiles from WT and bPACy strains under light and dark conditions. PCA was performed on log₂-transformed label-free quantification (LFQ) intensities obtained from biological triplicates of WT and bPACy strains under darkness or illumination conditions. The scores plot displays the first two principal components, with PC1 explaining 49.1% and PC2 explaining 23.3% of the total variance. Each point represents the mean of technical duplicates from one biological replicate; samples are colored by strain and shaped according to condition.

Next, we investigated the proteome in detail to analyze the effects of uncoupling glucose sensing from intracellular cAMP levels using optogenetic bPAC control (44). To elucidate the thereby caused differences between WT and bPACy, we first used dark conditions. Proteomic profiling was performed with a trapped ion mobility mass spectrometer (timsTOF) resulting in 1,883 protein abundances and 630 differentially abundant proteins (DAPs) (Fig. 3A; Table S1). Gene ontology (GO) term enrichment analysis revealed that 310 of the 630 DAPs matched to the generic GO term “catalytic activity,” with the main pathways being connected to ribosome biogenesis and RNA metabolism, and with additional upregulation of parts of the tricarboxylic acid cycle and changes in central metabolism pathways (Fig. 3A; Table S2). Moreover, Acs1, which produces acetyl-CoA as a precursor of β-carotene synthesis, was more abundant in the bPACy strain than in the WT strain under dark conditions (Table S1). Acs1 stands out with a roughly five-fold increased abundance, which was among the highest 10% abundance differences observed within the experiment. Additionally, in the biosynthesis pathway downstream of FPP toward ergosterol biosynthesis, the abundance of Erg1 was found to be increased in the bPACy strain compared to the WT strain under restrictive conditions, whereas the abundances of Erg4, Erg5, Erg28, and Erg29 were decreased. Interestingly, we found the low-affinity phosphodiesterase Pde1 downregulated in bPACy (dark) compared to WT (dark) (Table S1). The protein kinase Yak1 was found to be more abundant under these conditions, which is in agreement with the well-characterized changes in Yak1 abundance upon lowered PKA activity due to Msn2/4 activation (35, 45). Also, the Adr1 and Gis1 target genes Fox2 and Ape3 (respectively) were increased in the bPAC strain under dark conditions, which matches expectations for low PKA activity due to relieved inhibition of the transcription factors (35, 45).

Moreover, we also compared the metabolomes of WT and bPACy cells during proliferation-competent conditions of a light flux of 1 µmol m^−2^ s^−1^. Here, 2,190 proteins were detected above threshold, and 1,041 DAPs were identified. Again, we found DAPs mainly connected to ribosome biogenesis and other processes connected to RNA metabolism (Fig. 3B; Table S3). Again, we found the low-affinity phosphodiesterase Pde1 to be downregulated in bPACy cells under these conditions (Table S1).

Then, we compared the metabolome of bPACy cells in darkness with the metabolome of bPACy cells exposed to a blue light flux of 1 µmol m^−2^ s^−1^. We measured peptides of 2,058 proteins above threshold, and 759 DAPs were identified (Fig. 3C). Striking differences for the two conditions were connected to ribosome biogenesis and RNA metabolism (Fig. 3C; Table S4). We performed a clustering analysis of the proteomics results of all strains. As expected, this revealed a close relationship of the WT strain under both dark and light conditions, indicating that blue light exposure does not trigger a major stress response and causes only minor proteomic changes (Fig. S4). The proteome profile of bPACy under dark conditions is closer to the WT proteome than to the bPACy proteome under blue light conditions (Fig. 1C; Fig. S4). To gain more insight, a principal component analysis (PCA) was performed on log₂-transformed label-free quantification (LFQ) intensities obtained from biological triplicates of the WT and bPACy strains under dark or illuminated conditions (Fig. 3D). The plot displays the first two principal components, with PC1 explaining 49.1% and PC2 explaining 23.3% of the total variance. Separation along PC1 primarily distinguishes the two strains, indicating substantial strain-specific differences in global proteome composition. Along PC2, bPACy samples segregate according to illumination, with bPACy-L and bPACy-D forming distinct clusters, demonstrating a pronounced light-dependent proteomic response. In contrast, the WT replicates cluster closely together, regardless of illumination condition. Together, these results highlight the strong impact of optogenetic cAMP control on the bPACy proteome, while the WT background remains largely unaffected by the illumination regime.

**Fig 4:**
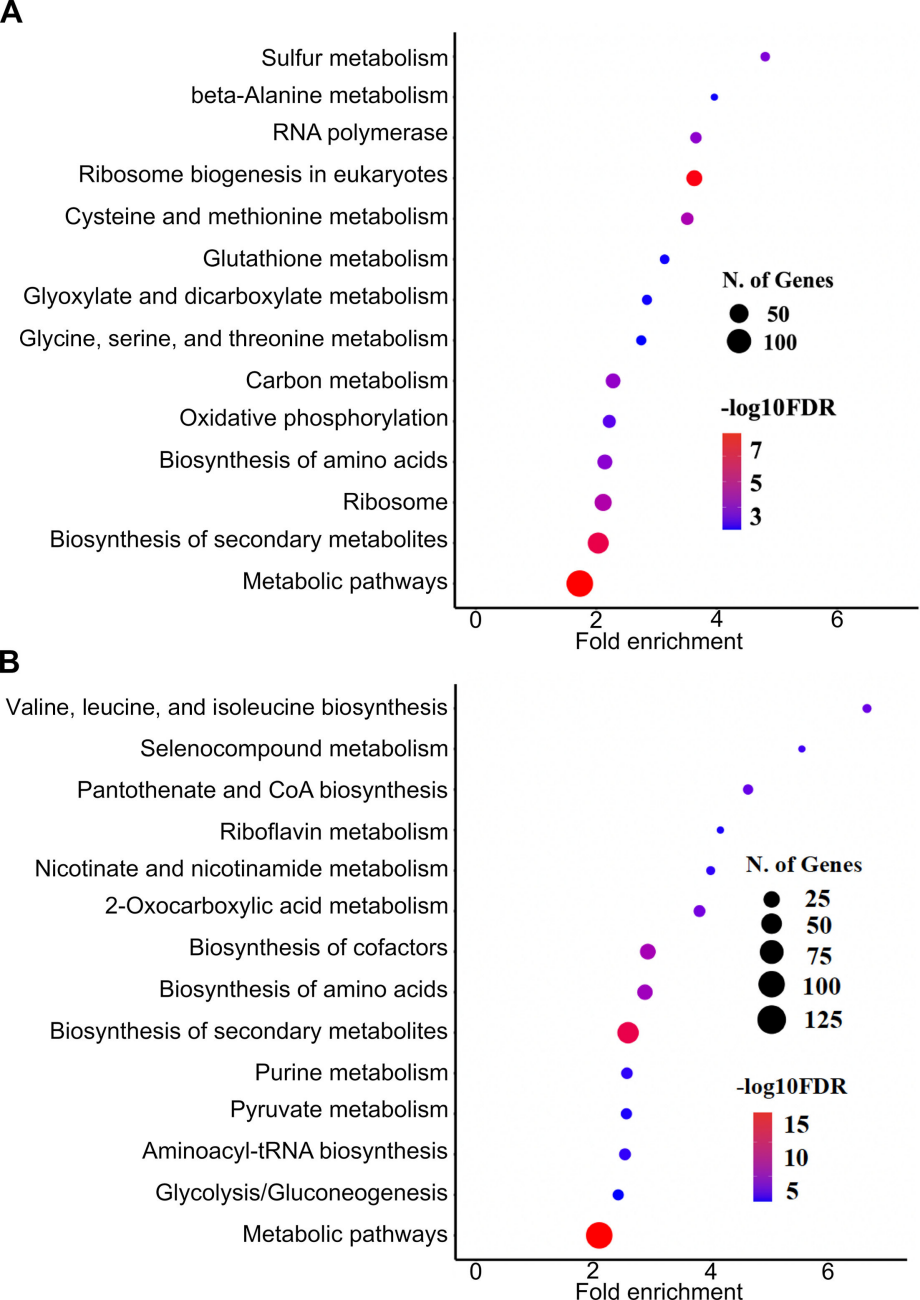
KEGG pathway analysis with DAPs obtained by a comparison of WT (dark) and bPACy (dark) using less stringent abundance criteria. (**A**) A list of proteins obtained with a less stringent abundance criterion (>20% abundance increase) was compared with the KEGG pathway database using ShinyGO. FDR cutoff was set to 0.05. (**B**) Same as in panel A, but a list of proteins with lower abundance (>20% abundance decrease) was used.

To assess the strain differences between bPACy and WT strains in higher detail, we compared protein abundance changes in darkness under less stringent conditions, i.e., at changes of a factor of 1.2 or higher, and compared them to the changes observable with an abundance change of 1.5 (Table S5). This revealed a higher number of changes in the central metabolism (Fig. S3A and B). Enzymes of the tricarboxylic acid cycle and the oxidative phosphorylation showed increased abundances. We mapped the 641 upregulated DAPs onto KEGG pathways and found 135 upregulated DAPs connected to metabolic pathways. The most striking pathways were again the ribosome and ribosome biogenesis, as well as carbon metabolism (Fig. 4A; Table S6). Similarly, we found 495 DAPs with lower abundances. Connected to metabolic pathways were 127 DAPs; the most striking pathways were purine and pyrimidine metabolism, as well as glycolysis/gluconeogenesis with its key enzymes of the central energy metabolism (Fig. 4B; Table S7).

**Fig 5:**
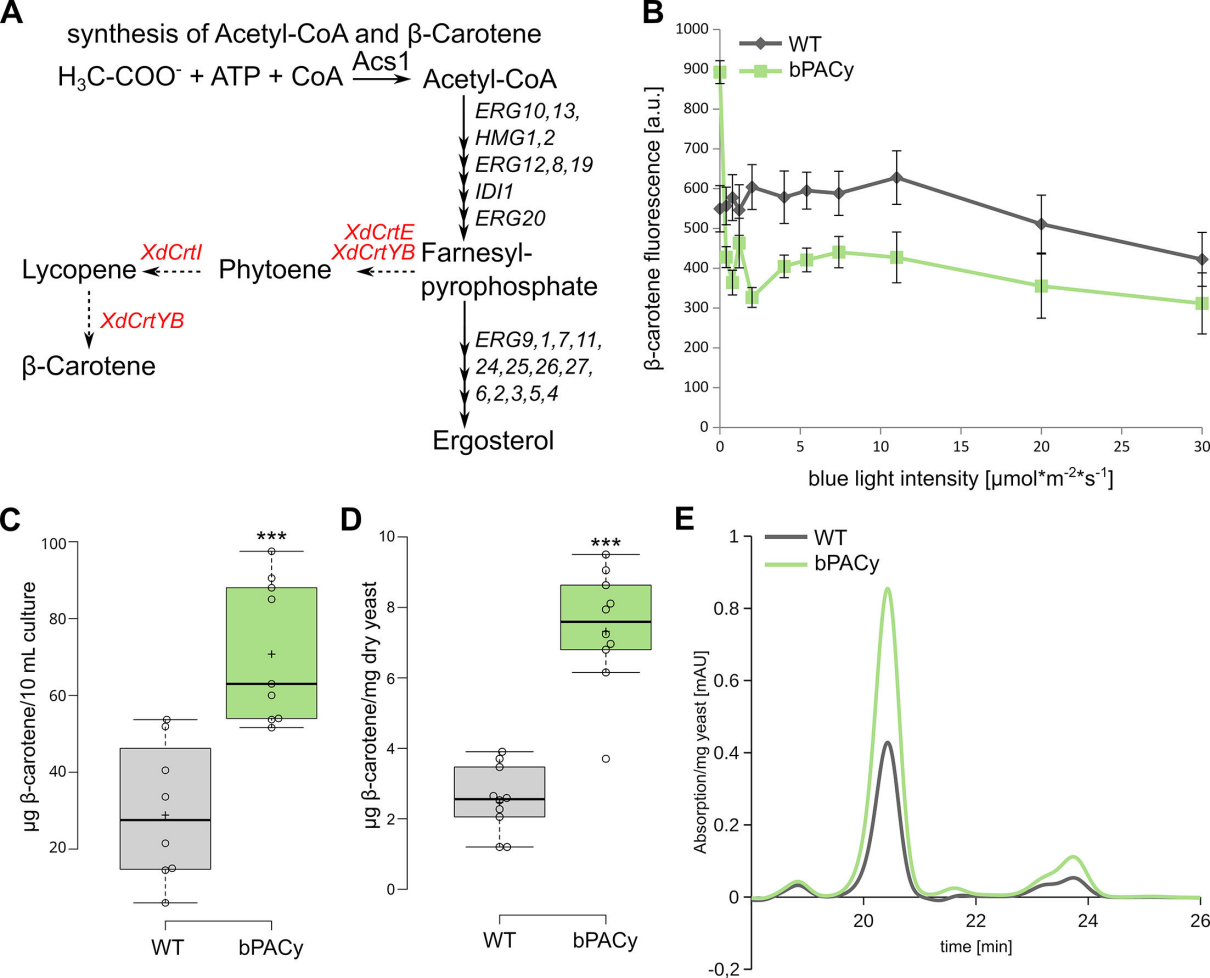
Increased β-carotene production by optogenetic control of protein kinase A (PKA) activity. (**A**) The heterologous β-carotene biosynthesis pathway from *Xanthophyllomyces dendrorhous* and its deviation from the yeast mevalonate pathway. The generation of Acetyl-CoA precursors by Acs1 using acetate, ATP, and coenzyme A (CoA) is indicated. (**B**) Dose-response curve for β-carotene production in WT (YCR75) and bPACy cells (YSEB38). Both strains were carrying pUDE269 expressing the β-carotene biosynthesis pathway from *Xanthophyllomyces dendrorhous*. Cells were grown overnight, illuminated with 1 µmol m^−2^ s^−1^ blue light (456 nm), diluted into fresh medium, and incubated for another 2 h with these conditions. Then, cells were illuminated with blue light (465 nm, light intensity as indicated) or incubated in darkness for 5 h before samples were taken. Flow cytometry was used to obtain the data (*n* = 5; error bars: standard error of the mean). (**C**) Biosynthesis of β-carotene in WT (YCR75) and bPACy cells (ySEB38) in darkness. Cells were grown for 24 h, illuminated with 1 µmol m^−2^ s^−1^ blue light (456 nm), and then shifted into darkness for another 24 h before samples were taken. The β-carotene amounts were quantified by HPLC analysis and normalized to culture volume (*n* = 9). Samples with known concentrations were used to quantify β-carotene amounts. (**D**) Measurement as in panel C, but normalized to yeast dry mass. (**E**) Diagram exemplifying β-carotene from WT and bPACy cells measured by HPLC. These are single measurements from the data shown in panel C.

Next, we investigated whether uncoupling of cAMP from glucose sensing favors the production of certain heterologous compounds. We used the production of the compound β-carotene as a model for the biosynthesis of heterologous products via the mevalonate pathway (Fig. 5A). Expression of the β-carotene pathway in bPACy and WT cells exposed to different illumination conditions revealed that the highest production levels were observed in bPACy cells incubated in darkness (Fig. 5B). Quantitative high-pressure liquid chromatography (HPLC) analysis revealed that we obtained more than twice the amount of β-carotene in the bPACy strain incubated in darkness compared to the corresponding WT strain when normalized against dry weight mass or culture volume (Fig. 5C through E). Please note that in both β-carotene experiments, a preceding growth phase under low amounts of blue light ensured the viability of the bPACy cells for the duration of the incubation period in darkness.

**Fig 6:**
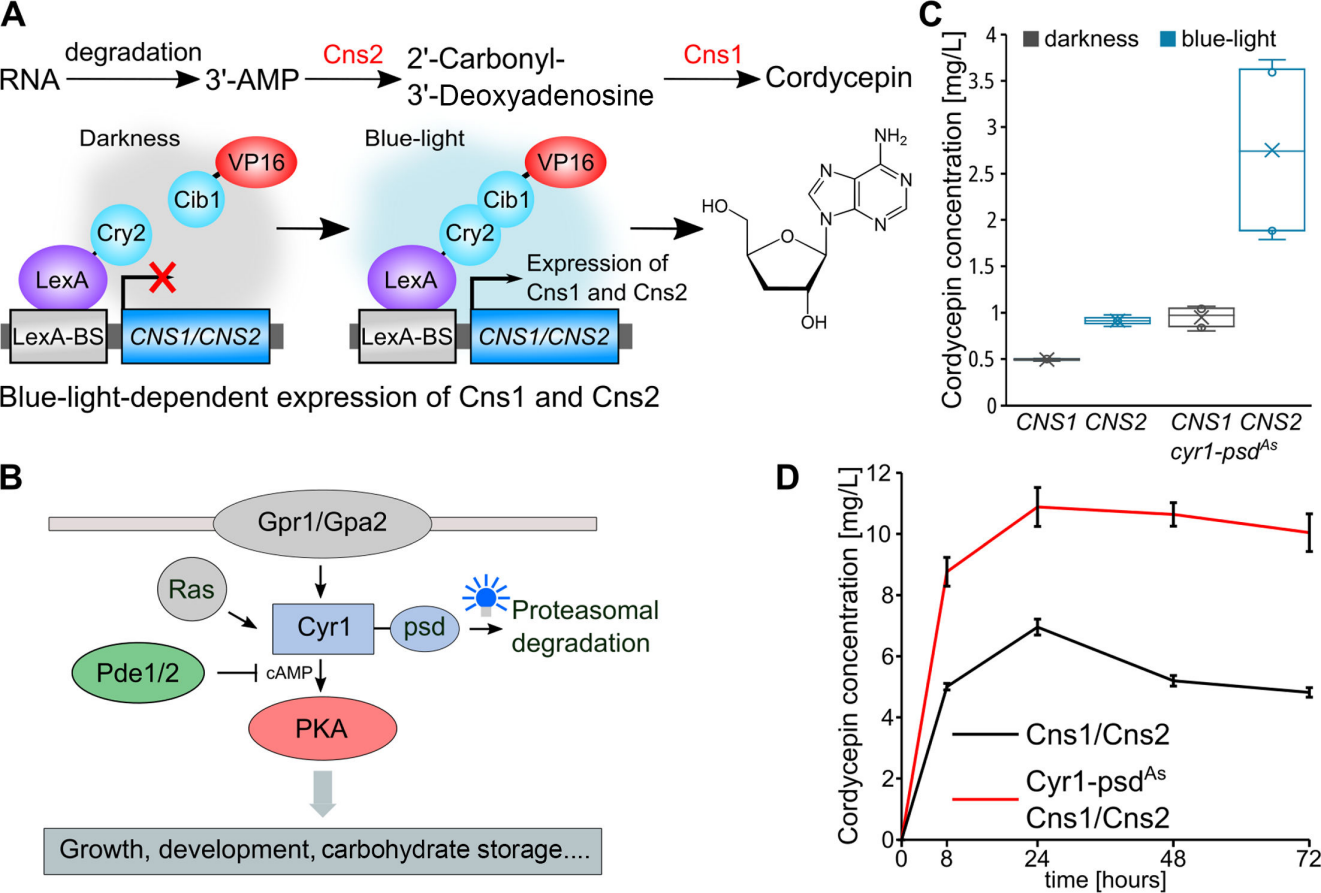
Characterization of cordycepin production in yeast. (**A**) Heterologous biosynthesis of cordycepin in yeast by expression of Cns1 and Cns2 from *Cordyceps militaris*. (**B**) Blue light-dependent destabilization of Cyr1 with a photo-sensitive degron (psd). (**C**) Quantification of cordycepin production after 24 h by yeast strains YBP3 (Cns1, Cns2) and YJT42 (*cyr1-psd^AS^*, Cns1, Cns2). Cordycepin was extracted from the culture medium (*n* = 4; error bars: standard error of the mean). Cells were grown for 12 h in darkness and then incubated for another 12 h in darkness or exposed to blue light (465 nm; 30 µmol s^−1^ m^−2^). (**D**) Quantification of cordycepin production over time. Cells were grown overnight in darkness and then incubated for 72 h exposed to blue light (465 nm; 30 µmol s^−1^ m^−2^). Samples were collected at the indicated time-points after the illumination shift (*n* = 3; error bars: standard deviation).

Next, we investigated whether production of other products can also be influenced by optogenetic manipulation of the PKA pathway. We implemented the biosynthesis pathway of cordycepin in yeast. The pathway starts with 3′-AMP, which is available through RNA degradation in *Saccharomyces cerevisiae,* and the enzymes Cns1 and Cns2 from *Cordyceps militaris* then produce cordycepin (Fig. 6A) (26, 46, 47). We overproduced Cns1 and Cns2 with a blue light-activated gene expression system (Fig. 6A) that was modified from an existing tool (26). Thus, we used a fully heterologous expression system that is not influenced by any regulatory mechanism inherent in yeast. To concomitantly achieve a reduction of cAMP levels and PKA activity, we added a photo-sensitive degron module to destabilize the yeast adenylyl cyclase Cyr1 upon blue light illumination (18, 48). Accordingly, blue-light illumination induces expression of Cns1 and Cns2 as well as a reduction in intracellular cAMP levels in this strain (Fig. 6A and B). Reduced PKA activity after blue light exposure of the cells was indicated by a darker color of light-exposed *cyr1-psd^AS^* cells due to iodine vapor staining compared to WT cells (Fig. S5A). Mass spectrometry analysis demonstrated that cordycepin production was increased in Cns1/Cns2-Cyr1-psd^AS^ cells exposed to blue light compared to the corresponding Cns1/Cns2 strain without light-dependent cAMP control (Fig. 6C). As expected, much lower amounts of cordycepin were produced in darkness. A time-resolved analysis of cordycepin production revealed that cordycepin production reached a plateau after 24 h; no further increase of cordycepin levels was found in cells of the Cns1/Cns2 strain or the Cns1/Cns2-Cyr1-psd^AS^ cells (Fig. 6C). The viability of the Cyr1-psd^AS^ cells was comparable with WT cells, although slightly reduced after 72 h exposed to blue light illumination. This prolonged blue light exposure also affected WT cell viability (Fig. S5B).

## DISCUSSION

Efforts to elevate intracellular cAMP levels in microbes have traditionally relied on activating endogenous adenylyl cyclases through tailored medium composition or specific cultivation conditions (49–51). In *Saccharomyces cerevisiae*, classical metabolic engineering has generated strains with up to 30-fold higher cAMP concentrations by targeting the PKA regulatory subunit Bcy1 and its catalytic subunits Tpk1, Tpk2, and Tpk3, thereby constitutively elevating PKA activity (52). PKA activity controls stress resistance in response to the availability of fermentable carbon sources, nitrogen, phosphate, osmotic stress, and the heat shock response (53–57). Accordingly, the cAMP–PKA axis of *Saccharomyces cerevisiae* has been recognized before as an attractive engineering target in biotechnology, e.g., in biofuel production utilizing xylose as carbon source from a regenerative feedstock under anaerobic fermentation conditions (58, 59). Besides implementing xylose metabolism genes, strain development required the additional manipulation of three intertwined signaling pathways that collectively govern growth and stress-resistance responses, namely the RAS/cAMP/PKA, Snf1, and high-osmolarity glycerol (HOG) pathways (60). During high carbon-intake processes such as biofuel production, *S. cerevisiae* cells encounter osmotic stress conditions imposed by high sugar concentrations. PKA modulates this response by controlling the stress-responsive transcription factor Msn2, yet the HOG pathway also influences this regulation (57). Moreover, industrial biofuel production requires robust thermotolerance, which depends on PKA activity as well (61, 62). Notably, hyperactive PKA signaling has been exploited in secondary fermentations for sparkling wine, where it promotes autolysis and flavor development (63).

In biotechnological contexts, precise exogenous control of the cAMP–PKA pathway has emerged as a desirable yet previously unattainable feature. The optogenetic platform presented here, placing the entire cAMP–PKA axis under direct, non-invasive photocontrol by bPAC, therefore addresses this task by providing dynamic, growth-phase-independent modulation of the cAMP–PKA axis. However, metabolic characterization of the bPACy strain revealed two striking features: firstly, the bPACy strain showed light-dependent increase of not only intracellular but also extracellular cAMP levels, which reach ~10- and ~90-fold increases for intra- and extracellular cAMP at high photon flux (10 µmol m^−2^ s^−1^), respectively (Fig. 2). Secondly, the bPACy strain exhibited, during the observation window, a low adenylate energy charge under all conditions, whether in darkness or in light. *S. cerevisiae* is renowned for its remarkable tolerance of low adenylate energy charge compared to most bacteria and higher eukaryotes, e.g., during the diauxic shift. The AEC levels in bPACy cells indicate a nutrient starvation-like state, despite being proliferation-competent when exposed to light and replete nutrients.

Synthetic manipulation of cAMP levels is not without its caveats, as a tool like bPAC can easily produce much more cAMP than is intracellularly necessary. Superfluous cAMP seems to be secreted from cells and may constitute a cAMP source for yeast that is used during lowered intracellular production.

According to our data, yeast growth relies on the intimate coupling between glycolysis/glucose availability and cAMP, but not on cAMP levels alone as observed in the bPACy strain. Notably, glycolysis in yeast cells is known to oscillate (64), where the oscillations with periods of about one minute are caused by allosteric regulation of phosphofructokinase. Besides being a potential pacemaker for other metabolic processes, cAMP oscillations can be induced by the fructose-1,6-bisphosphate/Cdc25/Ras/Cyr1 route (65). We suggest that this coupled cAMP dynamics is required for a detailed downstream balancing of energy metabolism and its involved enzymatic components. An absence of this coupling results in much stronger cAMP level variations over time and distorted growth behavior. Here, the protein kinase Snf1 may realize the feedback required for coupling, as Snf1 inhibits Cyr1 by phosphorylation (66) and is in turn inactivated by PKA via the protein kinase Sak1 (67). Despite belonging to the type of AMP-activated protein kinases, Snf1 is activated by ADP (68) as a signal for a low energy charge. This feedback between Snf1 and Cyr1 is broken in the bPACy strain and is expected to cause an overshooting Snf1 activation, which lowers the concentration of fructose-2,6-bisphosphate via phosphorylation-induced degradation of Pfk27 and thereby the glycolytic flux (69).

Finally, other non-cAMP-dependent effects on PKA, such as subcellular localization (70) and post-translational modifications, e.g., (auto)phosphorylation, also affect the cAMP–PKA axis, thus further complicating this regulatory network. Last but not least, different PKA activity levels cause differences in PKA target phosphorylation; thus, modulation of PKA activity transmits response changes (71).

Proteomic changes were found to underlie the AEC changes of the bPACy strain relative to wild type by a marked downregulation of all glycolysis enzymes except phosphoglucoisomerase, the latter not being subject to strong regulation in *S. cerevisiae* (72). Light exposure of bPACy emphasizes this downregulation relative to the dark state for several enzymes, e.g., for the gatekeeper gluco(hexo-)kinase isozymes of yeast. Interestingly, two minor isoforms of pyruvate decarboxylase, Pdc5 and Pdc6, are upregulated in bPACy. Pdc6 is part of the sulfur-sparing response of yeast due to its low cysteine/methionine content (73). The sulfur-sparing response is mediated by the master activator Met4, which is 34-fold increased in bPACy upon illumination but non-detectable in the wild-type strain. As part of this response, Met4 induces enzymes of the cysteine biosynthesis pathway (Fig. 3) and is transcriptionally upregulated by the starvation factor Gcn4 using a cAMP-dependent activation mode (74). Another indicator of a starvation-like state in bPACy that promotes availability of carbon sources in the cytosol is the lowered abundance of Fas1 and Fas2 and the α- and β-subunits of the fatty acid synthase, as well as the down- and upregulation of the mitochondrial/peroxisomal citrate synthases Cit1 and Cit2. Accordingly, other isozymes of the tricarboxylic acid cycle, such as succinyl-DH and isocitrate-DH, were found to be downregulated. Whereas the observed proteomic shifts in energy metabolism appear counterintuitive in bPACy-expressing cells, considering the elevated cAMP levels, ribosome biogenesis, an essential prerequisite for cellular growth, is clearly stimulated by the cAMP–PKA axis (Fig. 3). In concert with the TORC1 pathway (75), protein kinase A activity is known to promote the synthesis of ribosomal proteins and to coordinate ribosome biogenesis via control of the Fhl1/Ifh1/Crf1 complex in the nucleolus (76).

As counterparts of adenylyl cyclases, the cAMP-dependent phosphodiesterases Pde1 and Pde2 are crucial as an important regulatory part in PKA signaling. Hydrolysis of cAMP to AMP is required to decrease PKA activity after an activation burst through the Ras/cAMP/PKA pathway (32). Levels of the low-affinity phosphodiesterase 1 (K_M_ ~20–250 µM) are diminished in bPACy cells under dark and light conditions (Table S1). Unlike Pde1, which is under a very strong, nutrient-dependent control on the mRNA, but not protein level, the constitutive high-affinity Pde2 (K_M_ ~ 0.2 µM) controls the basal cAMP concentration. Here, PKA activity has been reported to affect Pde2 localization and overall protein levels (39). Interestingly, Pde2 levels in bPACy cells were also reduced by ~24% in darkness (Table S1). Overall, the PCA of bPACy and WT proteomes showed that the bPACy strain in darkness differs from the bPACy strain exposed to 1 µmol m^−2^ s^−1^, and both are different from the WT states, which cluster irrespective of the illumination conditions. This indicates that quite different PKA signaling states were implemented by replacing Cyr1 with bPAC.

As detailed above, the cAMP–PKA axis represents a highly promising target for engineering *Saccharomyces cerevisiae* cell factories toward enhanced heterologous overproduction of target compounds. Our findings reveal that decoupling this pathway from carbon metabolism, particularly from the glucose/glycolysis-derived signal transduced via Cyr1 and the Snf1-mediated feedback, triggered substantial changes. These can prove detrimental, as evidenced by reduced AEC, dysregulated sulfur metabolism, and impaired proliferative capacity. However, we obtained improved production of β-carotene and cordycepin, at least under specific conditions. This exemplifies the potential that synthetic control of the cAMP–PKA axis has for biotechnology applications.

In summary, our results demonstrate that the Cyr1-mediated integration of the cAMP–PKA axis into core carbon metabolism is essential for maintaining robust metabolic performance in *Saccharomyces cerevisiae*, which becomes markedly changed upon uncoupling of cAMP production from glucose sensing. Nevertheless, our results indicate that exactly under these conditions some heterologous compounds are produced in higher amounts. Further investigations will be required to elucidate the underlying mechanisms of this dynamic interplay, a phenomenon that cannot be fully captured by static, time-averaged cAMP measurements, as observed in the context of bPAC-mediated control.

## MATERIALS AND METHODS

### Yeast strains, growth conditions, and plasmids

All *Saccharomyces cerevisiae* strains were derivatives of the S288C strains, YJT23, and YJT24 (77), as well as the SK1 strains YCR75 and YCR76 (78). Strains are listed together with their relevant genotypes in Table S8. Standard preparations of media were used to grow cells (79). Low-fluorescence medium (LFM) was used to grow yeast cells in liquid cultures (80). Petri dishes were incubated at 30°C in darkness or exposed to blue light (465 nm, 30 µmol m^−2^ s^−1^). Chromosomal tagging of genes was performed with PCR products as described (81); yeast strains containing genes modified with the psd module were obtained similarly (82). The lithium acetate method was used for yeast transformations (83). Yeast cells were illuminated with blue light using high power LED stripes (465 nm; revoART, Borsdorf, Germany), StrawHat LED clusters (6 clusters of 42 LEDs, 465 nm; revoART, Borsdorf, Germany), or RGB LEDs (5,050 RGB LEDs; revoART, Borsdorf, Germany); each set equipped with a dimmer to select an appropriate light flux (0-30 μmol m^−2^ s^−1^). The light flux was checked before the experiment at the level of the yeast cells with an optometer (P2000, equipped with light-detector PD-9306-2, Gigahertz-Optik, Türkenfeld, Germany). Plasmids were constructed by standard procedures and are listed in Table S9; details and sequences of the used vectors are available on request.

### Flow cytometry measurements and statistics

Flow cytometry measurements were done as described in reference 26. Briefly, cells were grown in LFM medium to logarithmic growth phase, treated with sodium azide (10 µM end concentration) after sampling, diluted 1:10, and transferred to a multi-well plate. Fluorescence measurements were performed with an Attune NxT (ThermoFisher) equipped with an autosampler using a blue laser (488 nm) for excitation and to determine forward (FSC) and sideward scatter (SSC); a bandpass filter (530/30 nm) was used for detection of fluorescence in the green/yellow spectrum; a yellow laser (561 nm) was used for excitation; and a bandpass filter (620/15 nm) was used for fluorescence detection in the red spectrum. Height (FSC-H) versus area (FSC-A) plots were used to gate on single events. Additionally, gating was used to remove signals that accumulated on an axis. The fluorescence intensities were background-corrected using autofluorescence measurements of wild-type cells. Mean fluorescence measurements were used to assemble the graphs; graph generation was done with the software LibreOffice Calc; and error bars show the SEM or SD (as indicated in figure legends). Statistical analysis (two-sided unpaired Student’s *t*-test) was done with the software LibreOffice Calc.

### β-Carotene quantification from cell extracts by HPLC

β-Carotene quantification was done as described in reference 26. Briefly, *S. cerevisiae* cultures in 50 mL LFM medium were spun (2000 rpm, RT, 5 min), freeze-dried overnight in a Concentrator plus lyophilizer (*Eppendorf*), and weighed. The cells were lysed with glass beads in 1 mL of 50% MeOH, 50% acetonitrile (CAN) using a FastPrep-24 homogenizer (*MP* 370 *Biomedicals*, 6.5 m/s, 40 s). Suspensions were spun (13,000 rpm, RT, 5 min), and supernatants were transferred to fresh reaction cups. This process was repeated until the supernatant was colorless, and extracts from the same sample were pooled. Cell extracts were analyzed using an Agilent 1260 HPLC Infinity system (Agilent Technologies) equipped with an EC 250/4 NUCLEOSIL 100-5 C18 column (MACHEREY-NAGEL). From each sample, 25 μL was injected onto the column and separated using 50% MeOH and 50% ACN as eluent (1 mL/min, 25°C). β-Carotene was detected at λ = 450 nm and quantified using standards (*TCI*). Obtained chromatograms were processed with OpenLab CDS ChemStation Edition (Agilent Technologies).

### Cordycepin quantification from culture media samples by LC-MS/MS

Cordycepin quantification was done as described in reference 26. Briefly, 50 mL cultures were incubated for 12 h in darkness (30 °C, 90 rpm, 12 h) and afterward exposed to blue light (465 nm, 30 μmol m⁻² s⁻¹, 30 °C, 90 rpm, 12 h) or incubated further in darkness. Cultures were spun (4,000 rpm, 5 min), and 1 mL of each supernatant was heated (90°C, 10 min), spun (14,000 rpm, 5 min), and transferred to fresh reaction cups. Samples were analyzed using an LTQ-FT Ultra (Thermo Scientific) mass spectrometer equipped with an Agilent 384 1100 quaternary pump and an EC 150/2 NUCLEODUR 100-3 C18 (MACHEREY-NAGEL) column. Here, 20 μL was injected onto the column and separated by a linear gradient of ACN, 0.05% FA, and ddH2O, 0.05% FA (from 5 to 95% organic in 20 min). Cordycepin was detected in positive ion mode by the ICR cell at m/z = 252.1091 *±* 3 ppm and quantified using standards. Chromatograms were processed with Xcalibur v2.2 (Thermo Scientific).

### Proteomics sample preparation

WT and bPACy strain were inoculated to OD = 0.1 from an overnight culture and incubated for 12 h in LFM medium under blue light illumination (465 nm) at a flux rate of 1 μmol m^−2^ s⁻¹ (30°C, 80 rpm). After the initial growth phase, one set of cultures was shifted to arrest conditions (darkness) for 12 h, while the other set of cultures was incubated under blue light illumination (465 nm) at a flux rate of 1 μmol m^−2^ s⁻¹ for 12 h. The same medium was kept for the entire 24 h. After the 24-hour incubation period, the cells were harvested (2,000 rpm, 4°C, 10 min) and resuspended in 0.9% NaCl. One milliliter of each suspension was transferred to FastPrep vials and centrifuged (2,800 rpm, 4°C, 10 min). To the 8 M urea, 0.1 M NH_4_HCO_3_ was added for a total volume of 1 mL. Five hundred microliters of glass beads was added, and cells were lysed using a FastPrep-24 homogenizer (6.5 m/s, 6× 60 s, 2 min on ice after each run). The samples were spun to dissolve foam (14,000 rpm, 4°C, 45 min), and cell debris was gently resuspended. Full proteome peptide samples were prepared following an adaptation of a protocol applied for routine proteome analysis by the mass spectrometry facility of Philipps-University Marburg. Lysed cells equivalent to 200 μg protein were diluted with 8 M urea in 0.1 M NH_4_HCO_3_ to 40 μL, and pH was controlled for a value between 7 and 9. Samples were reduced with 1 μL of 0.2 M TCEP in 0.1 M NH_4_HCO_3_ using a thermal mixer (1,000 rpm, 37°C, 1 h) and alkylated in the dark with 1 μL of 0.4 M IAA (500 rpm, 25°C, 30 min). The reaction was quenched with 1 μL of 0.5 M NAC in 0.1 M NH_4_HCO_3_ (500 rpm, 25°C, 10 min), and samples were diluted with 10.3 μL of 0.1 M NH_4_HCO_3_ to 6 M urea. In preparation for proteolytic digestion, sample pH was controlled and adjusted to 8–9 if necessary. Alkylated protein samples were treated for 4 h at 37°C with protease Lys-C (2.5 μL, 0.2 μg/μL in 50 mM NH_4_HCO_3_, *m*_protease_/*m*_protein_ = 1/400), diluted with 145.2 μL of 0.1 M NH_4_HCO_3_, and treated O/N at 37°C with trypsin (4 μL, 0.5 μg/μL in 50 mM NH_4_HCO_3_, *m*_protease_/*m*_protein_ = 1/100). After adjusting the pH to <2 with TFA, the samples were centrifuged (13,000 rpm, RT, 1 min), and the supernatant was transferred to fresh reaction cups. Peptide samples were desalted using Chromabond C18 spin columns (100 μg peptide capacity) equilibrated with 150 μL ACN (2,000 rpm, RT, 30 s) and 3 × 150 µL 0.1% TFA in ddH_2_O (2,400 rpm, RT, 30 s). Columns were transferred to fresh reaction cups, and 100 μL sample solution diluted with 100 μL of 0.1% TFA in ddH_2_O was loaded onto the column (2,000 rpm, RT, 30 s). The flow-through was reapplied (2000 rpm, RT, 30 s), and bound peptides were washed with 3 × 150 µL of 5% ACN, 0.1% TFA in ddH_2_O (2400 rpm, RT, 30 s). Peptides were eluted into fresh reaction cups with 2 × 150 µL of 50% ACN, 0.1% TFA in ddH_2_O, dried at 45°C using a Concentrator plus vacuum centrifuge, and resuspended in 30 μL of 10% ACN, 0.1% TFA in ddH_2_O. Resulting peptide concentrations were determined using a NanoDrop spectrometer in 1 mg/mL mode, and concentrations were adjusted to 0.1 mg/mL with 10% ACN, 0.1% TFA in ddH_2_O for subsequent timsTOF analysis.

### Proteomics data analysis and visualization

The timsTOF data were analyzed using MaxQuant version 2.1.3. Protein sequence information for Saccharomyces cerevisiae (strain ATCC 204508/S288c; Baker’s yeast), comprising 6,742 entries, was retrieved from the UniProt database. For label-free quantification (LFQ) analysis, MaxQuant parameters were configured as follows: a maximum peptide mass of 4,000 Da, allowance of up to three modifications per peptide, up to three missed cleavages, requirement of MS/MS spectra for LFQ comparisons, and activation of the “match between runs” feature; all other parameters were kept at default settings. Protein abundances from biological triplicates of the wild-type and bPAC strains were compared using a two-tailed Student’s *t*-test (*P* < 0.05). Volcano plots were generated by plotting the log₂ fold change in protein abundance on the x-axis against the –log₁₀(*P*-value) on the y-axis. Plots were created in Python using the pandas library for data handling (84), NumPy for numerical computations (85), and matplotlib for figure generation (86). Automated label placement in volcano plots was achieved using the adjustText package (87) to visualize proteomic changes in relation to their statistical significance. Gene ontology (GO) enrichment analysis was performed on the sets of significantly changed proteins (*P* < 0.05) with log₂ fold change < −1 or > 1 from each comparison using the GOATOOLS library (version 1.2.3) (88). Statistical significance of the GO enrichment was assessed using the Benjamini–Hochberg procedure with a false discovery rate (FDR) threshold of *P* = 0.01. GO term annotations specific to *S. cerevisiae* (strain ATCC 204508/S288c) were used. Visualization of the GO analysis was carried out in *R*, where the x-axis represented the protein ratio, the y-axis represented the GO terms, bubble size indicated the number of proteins, and bubble color corresponded to the FDR *P*-value. Furthermore, the filtered list of proteins was mapped onto a metabolic network provided by the Kyoto Encyclopedia of Genes and Genomes (KEGG database) (89). A list of proteins generated with reduced stringency (1.2-fold abundance change, Student’s *t*-test *P* < 0.05) was used to assess metabolism shifts using ShinyGO (FDR cutoff 0.05) and KEGG database pathways as output (89, 90).

### Metabolomics sample preparation for the detection of cAMP, ATP, ADP, and AMP

The initial growth of the samples was performed as described for the proteomics sample preparation. For intracellular metabolite extraction, a protocol adapted from standard endometabolome analysis was used. Samples were taken under red-light illumination to avoid activation of bPAC by ambient light. One milliliter aliquots of yeast cultures were rapidly quenched by mixing with 1 mL of 70% methanol (vol/vol) pre-cooled to –80°C in pre-chilled 2 mL Eppendorf tubes, followed by centrifugation at 13,000 ×* g* for 10 min at –9°C to collect the cell pellets. After removal of the supernatant, pellets were stored at –80°C until extraction, which was carried out using a biphasic system consisting of methanol/TE buffer (1:1, vol/vol; 10 mM TRIS, 1 mM EDTA, pH 7.0) and chloroform, with extraction volumes adjusted based on biovolume calculations derived from OD₆₀₀ (e.g., 200 µL extraction fluid per 1 mL culture at OD₆₀₀ = 1), after which frozen pellets were resuspended in the calculated volumes of extraction fluid and chloroform, vortexed, incubated for 2 h at low temperature, and centrifuged (max speed, 10 minutes, –9°C, fixed-angle rotor) to separate the phases. The upper aqueous phase, containing polar metabolites such as cAMP, ATP, ADP, and AMP, was carefully collected, filtered, and stored at –80°C until LC-MS analysis.

For quantification of extracellular nucleotides, samples were collected at the same time as for intracellular samples. At each time point, 0.50 mL of culture was taken and immediately transferred to a pre-chilled 1.5 mL microtube, using a 0.2 µm syringe filter (Fisherbrand PTFE Syringe Filter) to remove cells. The resulting filtrates were stored at –80°C until LC–MS analysis.

### Viability assay by methylene blue staining

Cell viability was determined by methylene blue staining. Briefly, yeast strains were cultivated in LFM medium at 30°C and 80 rpm with or without illumination until sampling. Samples were collected and mixed 1:1 (vol/vol) with methylene blue solution (0.01% methylene blue, 2% sodium citrate) and incubated for 10 min at room temperature. Living and dead cells were counted using a Thoma counting chamber (depth 0.02 mm, grid area 0.0025 mm² per square) under a light microscope. Viable cells were identified as unstained (colorless), whereas non-viable cells appeared blue. Viability was calculated as the percentage of unstained cells relative to the total number of counted cells.

## Data Availability

The mass spectrometry proteomics data have been deposited to the ProteomeXchange Consortium via the PRIDE partner repository (91) with the dataset identifier PXD046730.

## SUPPLEMENTAL MATERIAL

Supplemental figures aem.02498-25-s0001.pdf Figures S1 to S5.

Table S1 aem.02498-25-s0002.xlsx Proteomics data.

Table S2 aem.02498-25-s0003.xlsx GO term analysis WT darkness vs bPACy darkness.

Table S3 aem.02498-25-s0004.xlsx GO term analysis WT vs bPACy, both strains exposed to a light flux of 1 µmol m^-2^ s^-1^ blue light (465 nm).

Table S4 aem.02498-25-s0005.xlsx GO term analysis bPACy strain incubated in darkness vs bPACy strain exposed to a light flux of 1 µmol m^-2^ s^-1^ blue light (465 nm).

Table S5 aem.02498-25-s0006.xlsx DAPs with higher and lower abundance (at least 1.2-fold change) WT (dark) vs bPACy (dark).

Table S6 aem.02498-25-s0007.xlsx KEGG pathway enrichment upregulated pathways WT (dark) vs bPACy (dark).

Table S7 aem.02498-25-s0008.xlsx KEGG pathway enrichment downregulated pathways WT (dark) vs bPACy (dark).

Table S8 aem.02498-25-s0009.docx Yeast strains used in this study.

Table S9 aem.02498-25-s0010.docx Plasmids used in this study.

ASM does not own the copyrights to Supplemental Material that may be linked to, or accessed through, an article. The authors have granted ASM a non-exclusive, world-wide license to publish the Supplemental Material files. Please contact the corresponding author directly for reuse.
