# Supplementary material for: Global impact on metabolic capacity of yeast cell factories by optogenetic control of the cAMP–PKA axis
Source: Appl Environ Microbiol. 2026 May 18;92(6):e02498-25. doi: 10.1128/aem.02498-25 (PMC13274398; doi:10.1128/aem.02498-25)
Supplement: Supplemental figures — Figures S1 to S5. [file aem.02498-25-s0001.pdf]

Supplemental Figures

Supplemental Figure 1

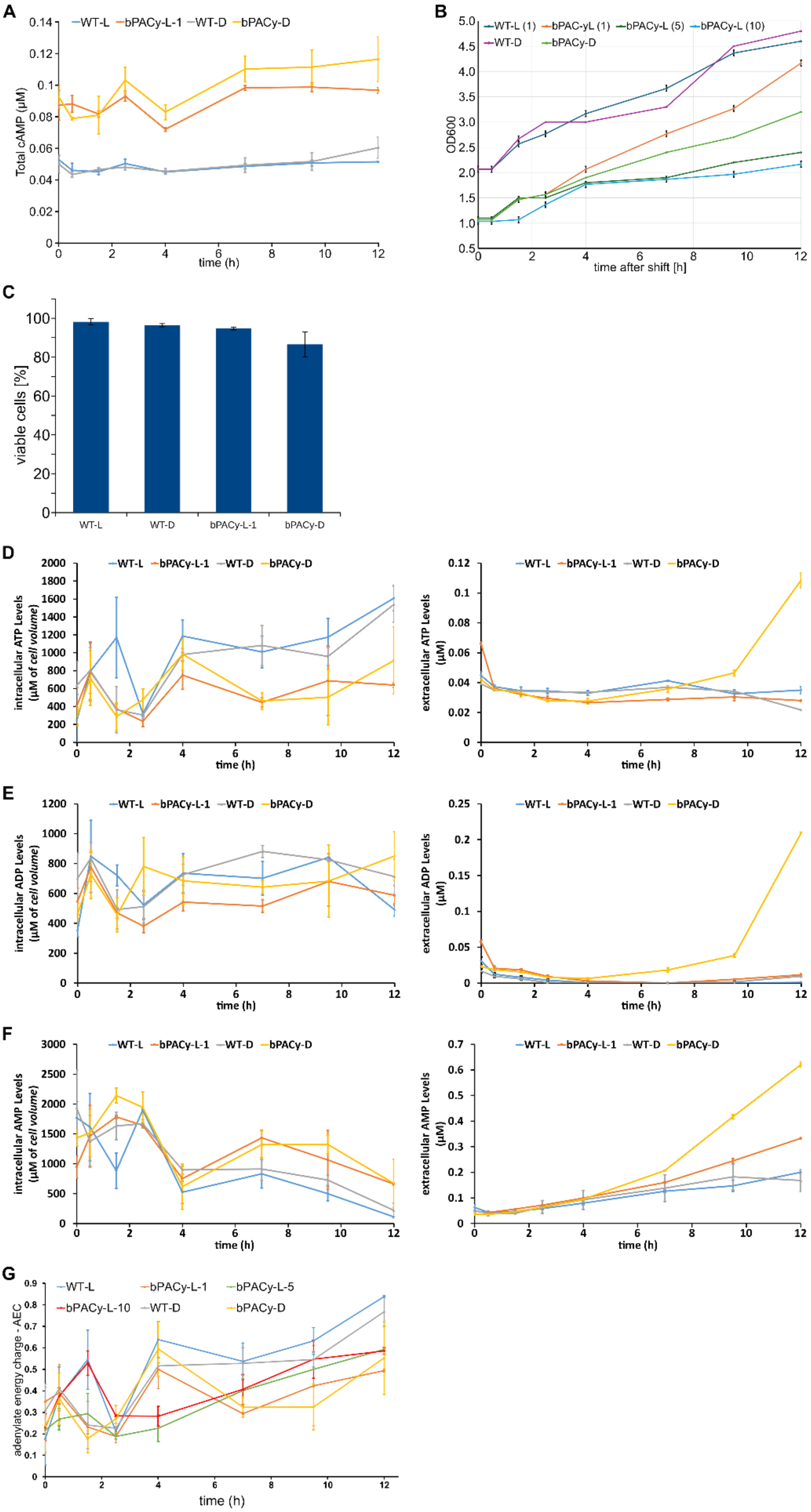

Supplemental Figure 1

A) Total cAMP concentration as sum of intracellular and extracellular cAMP levels over time in WT and bPACy cells incubated in darkness or exposed to a blue-light flux (465 nm) of 1 µmol m^{-2} s^{-1}. B) OD_{600} measurements of WT and bPACy strains. The measurements were performed during acquisition of the data shown in Figure 2, Supplementary Figures S1 A, E-G, and Supplementary Figure S2. (n=3; error bars standard deviation; please note that for some data points the variation is <0.1 OD_{600}) C) Viability staining with methylene blue of WT and bPACy cells after 24 h incubation time exposed to light conditions (1 µmol m^{-2} s^{-1}) or in darkness. Same experimental conditions as shown in Figure 2A. D-F) Intracellular and extracellular levels of ATP (B), ADP (C), and AMP (D) measured by liquid chromatography-mass spectrometry. G) The adenylate energy charge (AEC) was calculated to assess the cellular energetic state. The formular (ATP+0.5·ADP)/(ATP+ADP+AMP) was used to calculate the AEC. Data points are the mean of three independent biological replicates; error bars indicate standard deviation.

Supplemental Figure 2

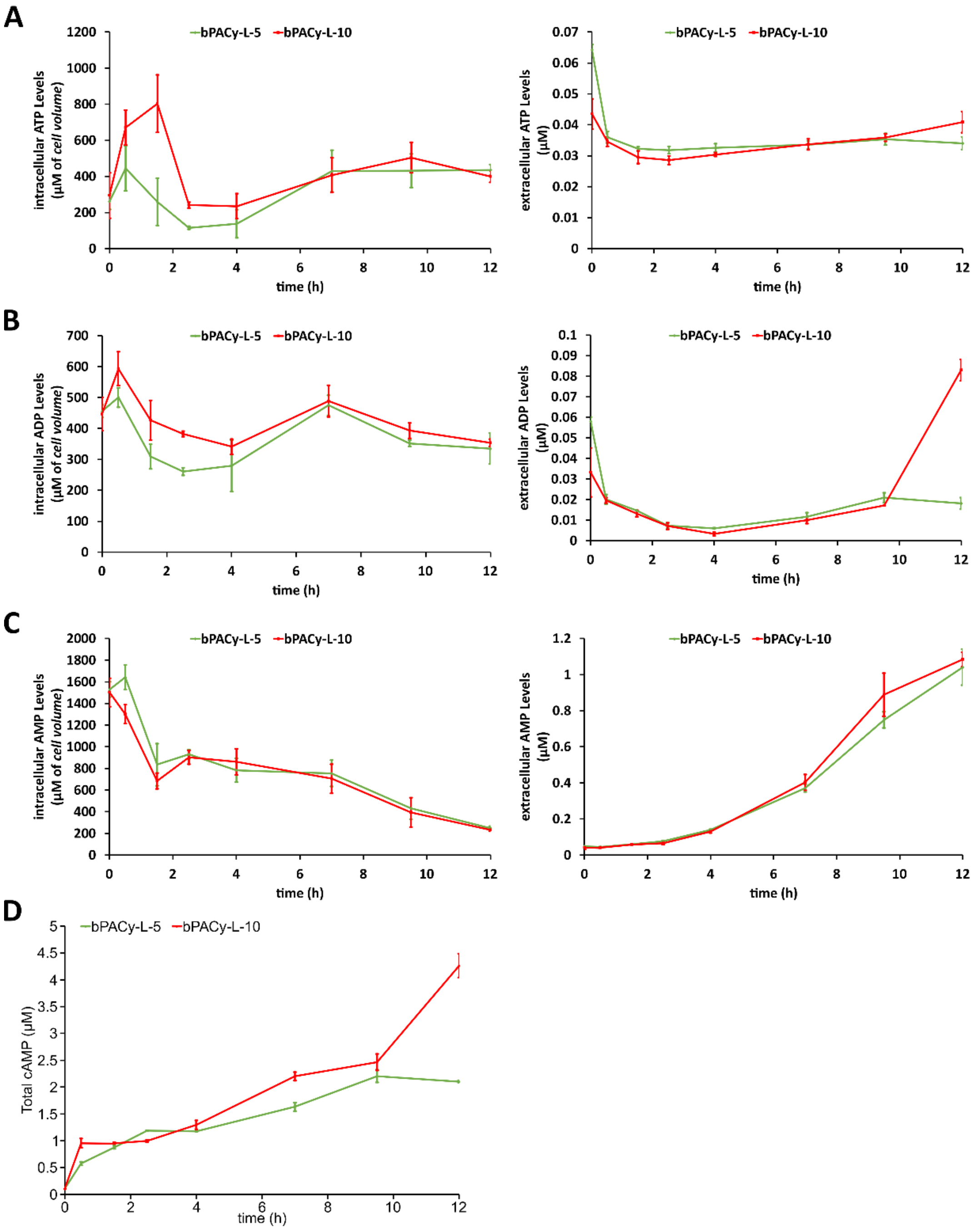

Supplemental Figure 2 Intracellular and extracellular adenylate nucleotide levels of bPACy cells. The bPACy cells were pre-cultured for 12 hours in LFM medium at 30 °C and 80 rpm under a blue-light flux (465 nm) of 1 µmol $\cdot m^{-2} \cdot s^{-1}$ and then shifted to a blue-light flux of 5 (bPACy-L-5) or 10 µmol m^{-2} s^{-1} (bPACy-L-10). Samples were taken at the indicated time points after the illumination shift. Intracellular (left graph) and extracellular (right graph) concentrations of ATP, ADP, and AMP are shown in A), B), and C), respectively. D) Total cAMP concentration as sum of intracellular and extracellular cAMP levels over time in bPACy cells exposed to a blue-light flux (465 nm) of 5 and 10 µmol m^{-2} s^{-1}. Nucleotide levels were quantified by LC-MS. Data points are the mean of three independent biological replicates (with the exception of bPACy-L-10 at 12 h, n=2); error bars indicate standard deviation.

Supplemental Figure 3

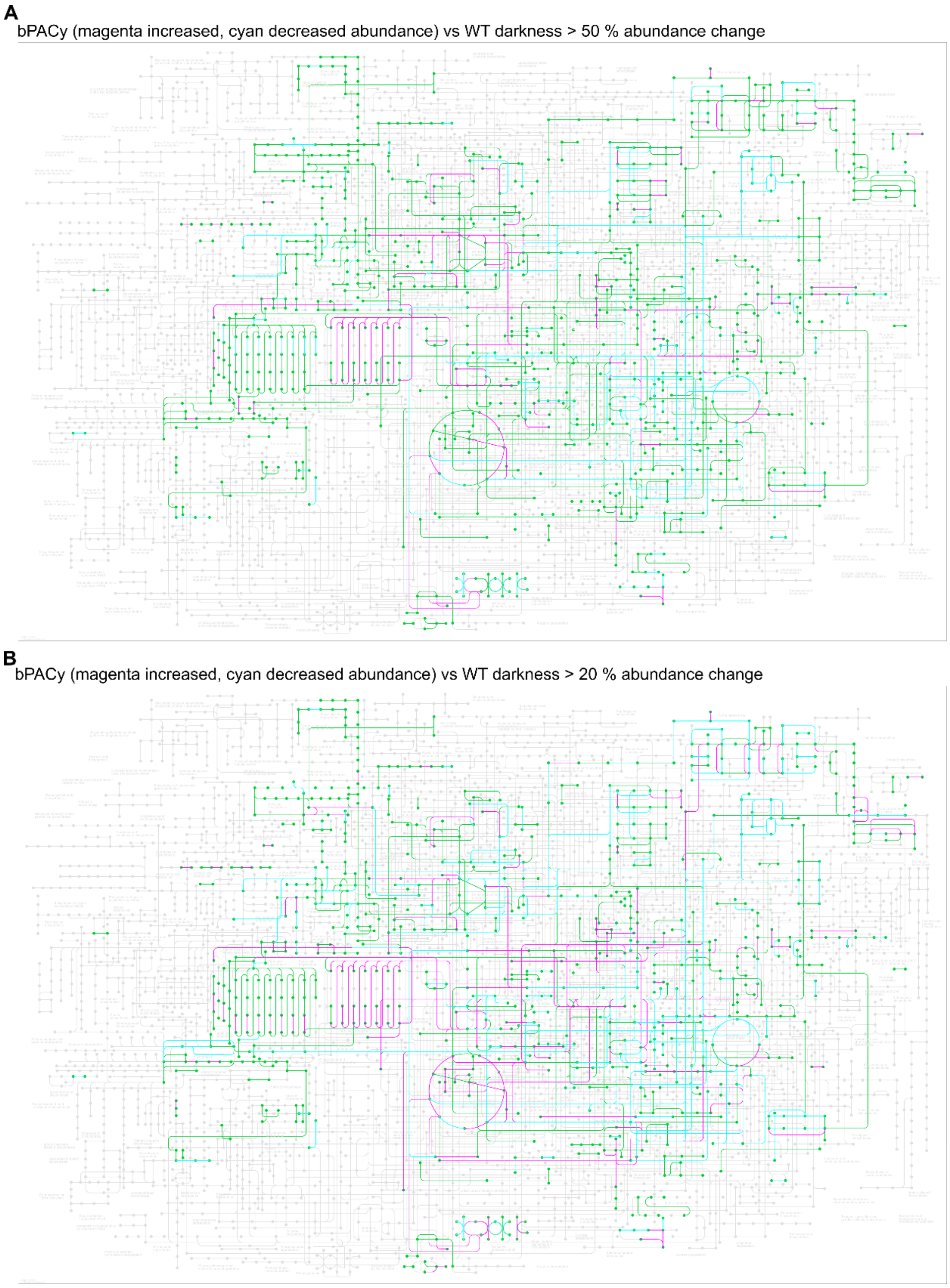

Supplemental Figure 3 Metabolic pathways affected by dynamic control of PKA activity in yeast

A) The filtered list of proteins from the WT (dark) versus bPACy (dark) condition (abundance change criterion > 1.5-fold) was mapped onto a metabolic network provided by the *Kyoto Encyclopedia of Genes and Genomes* (Kanehisa and Goto, 2000). Metabolic reactions in budding yeast are shown with green lines, upregulated reactions are highlighted with magenta lines, and downregulated reactions are depicted in cyan. The proteomics data used to obtain the filtered list is shown in **Figure 3**. B) same as in **A**, but the list of DAPs was obtained with a lowered abundance change (at least 1.2-fold).

Supplemental Figure S4

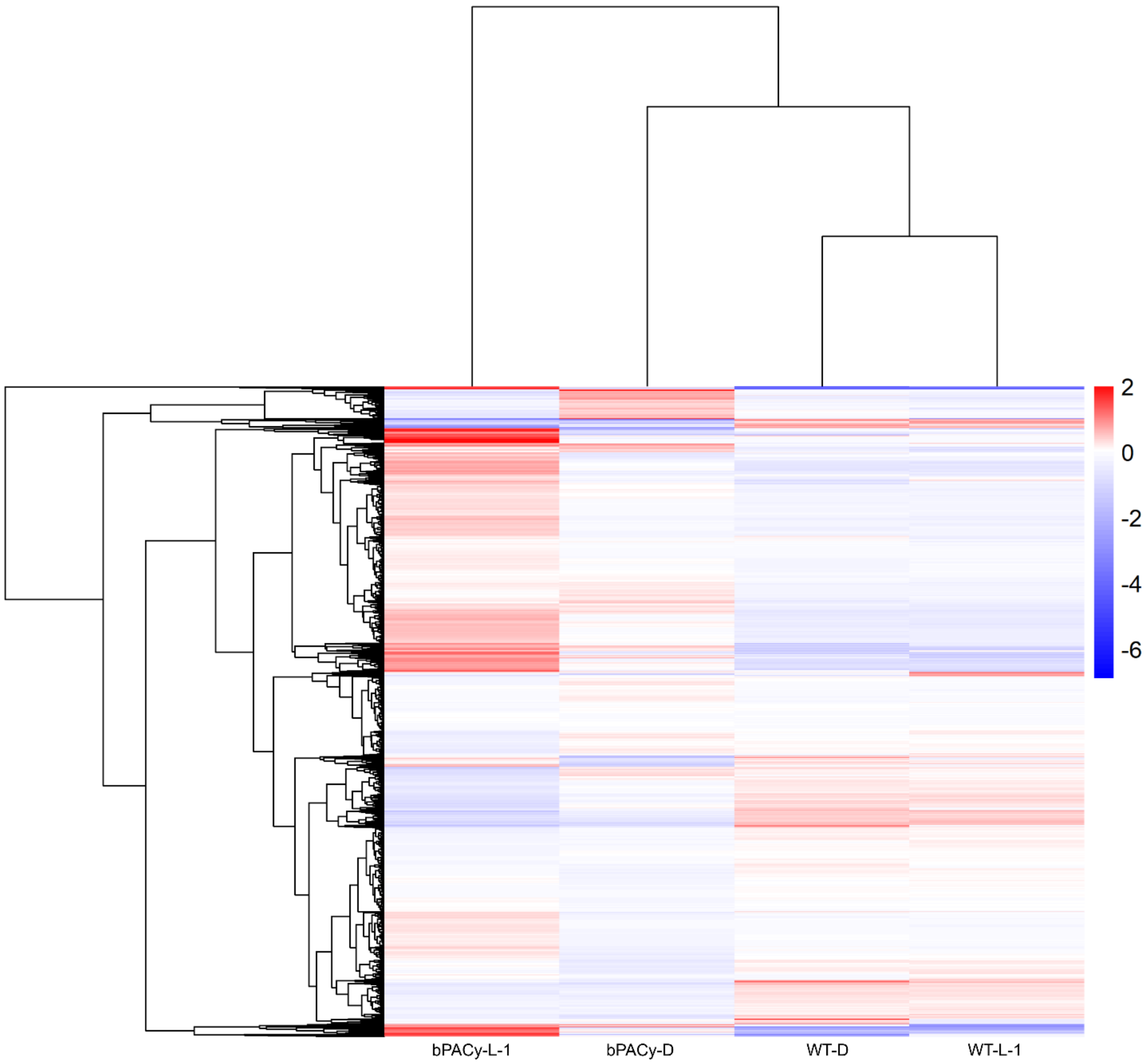

Supplemental Figure S4

Clustering analysis of proteomics results for the WT and bPAC strains incubated in darkness or under blue light (1 µmol m^{-2} s^{-1}). Abundance change of a protein in a sample in relation to the mean abundance is visualized.

Supplemental Figure 5

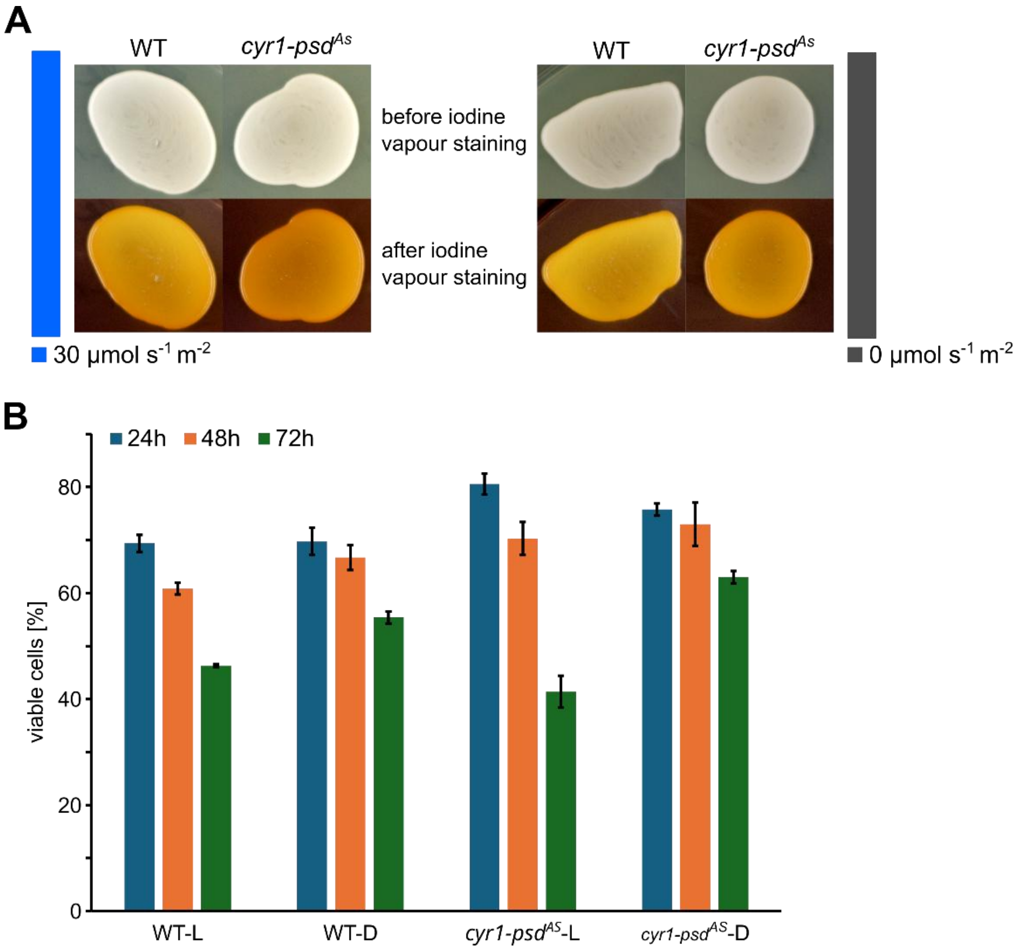

Supplemental Figure 5

A) Implementation of light-induced Cyr1 inactivation using the psd module. Iodine vapor staining of WT (YJT24) and *cyr1-psd*^{As} (YSH21) cells grown to patches on a plate exposed to a light flux of 30 µmol m^{-2} s^{-1} blue light (465 nm) or incubated in darkness. B) Methylene blue viability staining of WT (YJT24) and *cyr1-psd*^{AS} (YSH21) cells. Cells were exposed to the same conditions as for Figure 6D.
