# Supplementary material for: Global impact on metabolic capacity of yeast cell factories by optogenetic control of the cAMP–PKA axis
Source: Appl Environ Microbiol. 2026 May 18;92(6):e02498-25. doi: 10.1128/aem.02498-25 (PMC13274398; doi:10.1128/aem.02498-25)
Supplement: Table S8 — Yeast strains used in this study. [file aem.02498-25-s0009.docx]

**Supplementary Table S8: Yeast strains used in this study.**

| Name | Genotype | Source |
| --- | --- | --- |
| YCR75 | *MAT*α *his3 ura3 leu2 trp1 lys2 ho*::*LYS2* | (1) |
| YCR76 | *MAT*a *his3 ura3 leu2 trp1 lys2 ho*::*LYS2* | (2) |
| YJT23 | *MATα ura3-53 leu2Δ1 his3Δ200 trp1Δ63* | (3) |
| YJT24 | *MATa ura3-53 leu2Δ1 his3Δ200 trp1Δ63* | (3) |
| YSEB38 | YCR75 *cyr1Δ::hphNT1, pDS312 [*P*_CYC1_-tagRFP-bPAC ARS/CEN LEU2]* | (4) |
| YBP3 | YJT23*, MATα ura3-53 leu2Δ1, trp1Δ63, his3::hphNT1::P_ADH1_-VP16-CIB1::his3 ura3::natNT2::P_ADH1_-LexA-BD-Cry2::ura3 leu2::P_LexA_-myCitrine-Cns2-T_DIT1_::leu2 [pDS261: P_LexA_-myCitrine-Cns1-T_DIT1_ CEN/ARS URA3]* | (5) |
| YJT42 | YBP3 *his3::hphNT1::P_ADH1_-VP16-CIB1::his3 ura3::natNT2::P_ADH1_-LexA-BD-Cry2::ura3 leu2::LexA-myCitrine-Cns2::leu2 cyr1-3myc-psd^AS^::kanMX6 [pDS261: P_LexA_-myCitrine-Cns1-T_DIT1_ CEN/ARS URA3]* | This study |
| YSH21 | YJT24 *cyr1-3myc-psd^AS^::kanMX6* | This study |

**References**

1. Renicke, C., Allmann, A. K., Lutz, A. P., Heimerl, T., and Taxis, C. (2017) The mitotic exit network regulates spindle pole body selection during sporulation of Saccharomyces cerevisiae. *Genetics*. **206**, 919–937

2. Lutz, A. P., Schladebeck, S., Renicke, C., Spadaccini, R., Mösch, H. U., and Taxis, C. (2018) Proteasome activity is influenced by the HECT_2 protein Ipa1 in budding yeast. *Genetics*. **209**, 157–171

3. Pook, B., Goenrich, J., Hasenjäger, S., Essen, L.-O., Spadaccini, R., and Taxis, C. (2021) An Optogenetic Toolbox for Synergistic Regulation of Protein Abundance. *ACS Synth. Biol.* 10.1021/ACSSYNBIO.1C00350

4. Hepp, S., Trauth, J., Hasenjäger, S., Bezold, F., Essen, L.-O., and Taxis, C. (2020) An optogenetic tool for induced protein stabilization based on the Phaeodactylum tricornutum aureochrome 1a LOV domain. *J. Mol. Biol.* 10.1016/j.jmb.2020.02.019

5. Bezold, F., Scheffer, J., Wendering, P., Razaghi-Moghadam, Z., Trauth, J., Pook, B., Nußhär, H., Hasenjäger, S., Nikoloski, Z., Essen, L. O., and Taxis, C. (2023) Optogenetic control of Cdc48 for dynamic metabolic engineering in yeast. *Metab. Eng.* **79**, 97–107
