# Supplementary material for: Global impact on metabolic capacity of yeast cell factories by optogenetic control of the cAMP–PKA axis
Source: Appl Environ Microbiol. 2026 May 18;92(6):e02498-25. doi: 10.1128/aem.02498-25 (PMC13274398; doi:10.1128/aem.02498-25)
Supplement: Table S9 — Plasmids used in this study. [file aem.02498-25-s0010.docx]

**Supplementary Table S9: Plasmids used in this study.**

| Name | Genotype | Source |
| --- | --- | --- |
| pRS315 | LEU2 CEN/ARS ori bla | (1) |
| pRS316 | URA3 CEN/ARS ori bla | (1) |
| pDS312 | pRS315 P*_CYC1_-tagRFP-bPAC* | (2) |
| pUDE269 | pTDH3-crtYB-T2A1-crtI-T2A2-crtE-tTEF 2µ URA3 ori bla | (3) |
| pDS261 | pRS316 *P_LexA_-YFP-CNS1-T_DIT1_* | (4) |

**References**

1. Sikorski, R. S., and Hieter, P. (1989) A system of shuttle vectors and yeast host strains designed for efficient manipulation of DNA in Saccharomyces cerevisiae. *Genetics*. **122**, 19–27

2. Hepp, S., Trauth, J., Hasenjäger, S., Bezold, F., Essen, L.-O., and Taxis, C. (2020) An optogenetic tool for induced protein stabilization based on the Phaeodactylum tricornutum aureochrome 1a LOV domain. *J. Mol. Biol.* 10.1016/j.jmb.2020.02.019

3. Beekwilder, J., van Rossum, H. M., Koopman, F., Sonntag, F., Buchhaupt, M., Schrader, J., Hall, R. D., Bosch, D., Pronk, J. T., van Maris, A. J. A., and Daran, J. M. (2014) Polycistronic expression of a β-carotene biosynthetic pathway in Saccharomyces cerevisiae coupled to β-ionone production. *J. Biotechnol.* **192**, 383–392

4. Bezold, F., Scheffer, J., Wendering, P., Razaghi-Moghadam, Z., Trauth, J., Pook, B., Nußhär, H., Hasenjäger, S., Nikoloski, Z., Essen, L. O., and Taxis, C. (2023) Optogenetic control of Cdc48 for dynamic metabolic engineering in yeast. *Metab. Eng.* **79**, 97–107
